# CircRNA_01754 Regulates Milk Fat Production Through the Hippo Signaling Pathway

**DOI:** 10.3390/ani15243606

**Published:** 2025-12-15

**Authors:** Xiaofen Li, Jiahao Chen, Rui Gao, Ye Feng, Zhifeng Zhang, Zhi Chen

**Affiliations:** 1 School of Animal Science and Technology, Jiangsu Agri-Animal Husbandry Vocational College, Taizhou 225300, China; 2 College of Animal Science and Technology, Yangzhou University, Yangzhou 225009, China

**Keywords:** LATS2, circRNA_01754, miR-302c, Hippo signaling pathway, milk fat metabolism

## Abstract

Background: Milk fat metabolism is a complex process regulated by non-coding RNAs. circRNAs (circRNAs) can act as miRNA sponges to regulate target gene expression. Purpose: This study aimed to investigate the role and mechanism of the circRNA_01754/miR-302c/LATS2 axis in regulating milk fat metabolism in bovine mammary epithelial cells (BMECs). Methods: mRNA sequencing was performed on bovine mammary gland tissues from different lactation stages to analyze the expression profiles. The interactions between circRNA_01754, miR-302c, and LATS2 were verified using dual-luciferase reporter assays, qRT-PCR, and Western blot. The functional effects on triglyceride (TAG) and cholesterol synthesis were assessed. Results: CircRNA_01754 was identified as a direct sponge for miR-302c. Overexpression of circRNA_01754 increased LATS2 expression by sequestering miR-302c. Functional experiments showed that circRNA_01754 and LATS2 promoted TAG synthesis, whereas miR-302c inhibited it. Furthermore, miR-302c was found to regulate the expression of YAP1, a key effector of the Hippo pathway, through LATS2. Conclusions: Our findings reveal that circRNA_01754 promotes milk fat synthesis by acting as a ceRNA for miR-302c to upregulate LATS2. This study lays the groundwork for producing high-quality milk and opens up new avenues for enhancing public dietary health.

## Simple Summary

Fat content is a key factor determining the nutritional value and quality of milk. However, the molecular mechanisms controlling milk fat synthesis are not fully understood. This study investigated how a circular RNA molecule called circRNA_01754 affects fat production in cow mammary cells. We found that circRNA_01754 acts like a sponge by binding to a small molecule named miR-302c. This binding triggers the inhibition of a target gene called LATS2, which is part of the Hippo signaling pathway. Increasing the levels of either circRNA_01754 or LATS2 promoted the synthesis of triglycerides, the main component of milk fat, while increasing miR-302c had the opposite effect. These findings reveal a novel regulatory pathway involving circRNA_01754, miR-302c, and LATS2 in controlling milk fat metabolism. This study provides important insights for future strategies aimed at improving milk quality and developing healthier dairy products through molecular breeding.

## 1. Introduction

The completion of the Human Genome Project has attracted scientists’ attention to the function and role of non-coding RNAs, which account for about 98% of the gene coding sequence [1,2,3]. Sharing the same characteristics, non-coding RNAs are transcribed from the genome and play a key role in the regulation of gene expression. In general, they regulate the process of gene transcription and translation; however, they do not translate into proteins, and thus, they only function at the RNA level [4]. In recent years, there has been continuous research in this field; growing numbers of researchers believe that non-coding RNAs play indispensable roles in the gene-encoding protein, and they have a close relationship with physiology, disease, and other aspects of the body [5].The function and mechanisms of action of non-coding RNAs in prostatic diseases. A typical example is breast lactation, which is precisely regulated by hormones and genes in the body. miRNAs regulate many lactation-related genes in this complex regulatory network. They play an important role in the post-transcriptional regulation of fatty acids and their metabolism-related genes and participate in the synthesis and decomposition of fatty acids [6]. Here, we explore the molecular mechanism of miRNAs’ co-regulation of milk fat metabolism in dairy cows and construct the regulatory network between miRNA genes to provide a theoretical and experimental basis for the study of the milk fat metabolism mechanism in dairy cows. There are a large number of miRNA binding sites on circRNA, which can serve as endogenous RNA to compete with mRNA for miRNA. By binding with miRNA, they exert a sponge-like effect and regulate the expression of target genes [7,8]. Research has shown that miR-378a-3p can target and bind to HDAC4 in Qinchuan cattle, inhibiting the proliferation of bovine skeletal muscle cells and promoting their differentiation and apoptosis. CircLMO7 competitively binds to miR-378a-3p with HDAC4 to regulate the growth and development of bovine skeletal muscle [9]. These research results show that circular RNA (circRNA) is a type of covalently closed circular RNA, rich in microRNA (miRNA)-binding sites, which can lead to miRNA’s inhibition of messenger RNA (mRNA) and increase its expression level. This circRNA–miRNA–mRNA regulatory network is also known as the competitive endogenous RNA (ceRNA) network [10]. In this study, we screened for differentially expressed mRNAs during lactation and constructed a core ceRNA (ircRNA–miRNA–mRNA) network to elucidate the molecular mechanisms underlying the occurrence and development of lactation in cows, providing experimental evidence for producing high-quality milk.

In 1995, Justice et al. applied genetic chimerism screening technology and discovered that the mutation of the warts gene (tumor suppressor gene) would cause the excessive growth of Drosophila melanogaster [11]. The Hippo pathway also exists in mammals, primarily regulating organ size during development. More specifically, it regulates some downstream target genes through the phosphorylation and inactivation of transcription factors caused by a series of kinase cascade reactions [12]. Recent research has focused attention on the Hippo signaling pathway and lipid metabolism since they are involved in the regulation of fat development and differentiation [13]. Specifically, the activation of important signal molecules upstream of the Hippo pathway results in a reduction in neurofibromin 2 (NF2) activity, thereby causing a series of cascade reactions of key factors: Mst1/2 is activated, Lats1/2 is phosphorylated and activated simultaneously, and the activation of Lats1/2 further phosphorylates YAP. Therefore, it is imperative to understand the function and regulatory pathway of LATS2 in the study of the Hippo signaling pathway. In addition, the activity of YAP decreases significantly after phosphorylation and remains in the cytoplasm. Nevertheless, p-YAP and P-TAZ are locked from entering the nucleus and combining with TEAD, resulting in TEAD losing its transcriptional activity. This is equivalent to the inhibition of the Hippo pathway [14,15].

## 2. Materials and Methods

### 2.1. Ethical Statement

The Animal Use Agreement in this experiment was approved by the Animal Protection and Utilization Committee of the School of Animal Science and Technology of Yangzhou University.

### 2.2. Collection of Breast Tissue Samples

This study used healthy and disease-free Holstein cows raised on the dairy farm of Yangzhou University as experimental animals. All the cows were thirdborns and were raised in the same environment from birth until the collection of mammary gland samples. They were fed freely and exposed to natural light, and mammary gland tissue was collected during the early lactation and peak lactation periods. A surgical method was used to harvest the breast tissue, which was immediately placed in RNA-free cryovials and stored in liquid nitrogen. The experiment was performed with three biological replicates.

### 2.3. RNA Extraction and Sequencing Analysis

Total RNA was extracted from the breast tissue using the RNAiso Plus assay kit (TaKaRa, Dalian, China). We used NanoDrop 2000 (Thermo Fisher Scientific, Waltham, MA, USA) to analyze the RNA quality, and RNA samples were only used for subsequent sequencing analysis when the RNA integrity value was greater than 8. After generating the raw data, the sequencing quality was evaluated using FastQC (0.12.0)software, and low expression genes were filtered, based on fragment values per kilobase transcript read per million mappings. We performed differential expression gene (DEG) analysis using DESeq2 (1.44.0) software. The experiment analyzed the differentially expressed genes (DEGs) between different comparison groups and displayed them using volcano plots and expression heatmaps. We conducted gene function annotation on the DEGs identified in each comparison using the GO database, followed by a KEGG signaling pathway enrichment analysis on these genes. The specific enrichment analysis was implemented using the R package (4.3.1) clusterProfiler.

### 2.4. Cell Culture

BMECs were cultured and propagated in the basal medium containing 90% DMEM/F12 (Gibco, Thermo Fisher Scientific, Waltham, MA, USA), 10% fetal bovine serum (Gibco, Thermo Fisher Scientific, Waltham, MA, USA), 100 U/mL penicillin/streptomycin, 5 µg/mL insulin, 1 µg/mL hydrocortisone, 10 ng/mL growth factor, and 2 µg/mL prolactin (Sigma-Aldrich, St. Louis, MO, USA). Small RNAs were transfected by the reverse transfection method recommended in the instructions of Invitrogen’s (Waltham, MA, USA) Lipofectamine RNAiMAX Reagent transfection reagent, and the final concentration of small RNAs was 60 nM. The miR-302c mimic, miR-302c inhibitor, siRNA-LATS2, and siRNA-YAP1 used in this study were synthesized by GenePharma (Shanghai, China). The siRNA sequences are provided in the Supplementary Materials (Supplementary Table S1). The BMECs were obtained from the Bovine Genetic Resources Laboratory at the College of Animal Science and Technology, Yangzhou University.

### 2.5. Plasmid Construction and Cell Transfection

The full-length sequence of circRNA_01754 was amplified using bovine mammary gland cDNA as the template. For the overexpression plasmid, HindIII and KpnI restriction sites were introduced at the ends of the amplified product, which was then cloned into the pcDNA3.1 vector to generate pcDNA-circRNA_01754.

For the dual-luciferase reporter assay, a wild-type vector containing the predicted miR-302c binding site was constructed. The circRNA_01754 sequence was amplified with XhoI and NotI restriction sites at its ends and inserted into the psiCHECK-2 vector to create the psiCHECK-2-circRNA_01754-wild-type (WT) reporter plasmid (Supplementary Table S2).

For transfection, the constructed psiCHECK-2-circRNA_01754-WT reporter plasmid and the miR-302c mimic were co-transfected into HEK293T cells using a liposome-based transfection method. HEK293T cells were chosen for their high transfection efficiency and stability in reporter gene assays, making them a standard model for validating miRNA–circRNA interactions. Transfection was performed when the cells reached approximately 75% confluence in 48-well plates. Cells were harvested 48 h post-transfection, and the luciferase activity was measured.

### 2.6. Real Time Fluorescence Quantification

The TRIzol kit (TaKaRa, Dalian, China) was used to extract total RNA from the samples. The reverse transcription reaction was assembled in a total volume of 10 μL, containing 500 ng of total RNA, 2 μL of 5 × Mix, 0.5 μL of Random6 primers, 0.5 μL of oligo dT primers, and nuclease-free water to volume. The reaction was conducted under the following conditions: 37 °C for 15 min, 85 °C for 5 s, and a final hold at 4 °C. Real-time quantitative PCR was performed using a Bio-Rad CFX96 system. The total reaction volume of 25 μL consisted of 12.5 μL of SYBR Premix Ex Taq II (Q712; Vazyme, Nanjing, China), 10 ng of cDNA, 10 μmol/L each of the forward and reverse primers, and it was brought to volume with ddH_2_O. The qRT-PCR conditions were as follows: 95 °C for 30 s, 39 cycles of 95 °C for 5 s and 60 °C for 30 s, and 95 °C for 10 s and 65 °C for 5 s. The relative abundance was calculated using the 2^−ΔΔCT^ method, with β-actin as an internal control gene. The experiment was performed with three biological and three technical replicates.

### 2.7. Western Blot

After treating cells via protein extraction in a 60 mm culture dish for 48 h, the culture medium was discarded. Then, the cells were washed twice with PBS before being placed in an ice bath to completely remove the culture medium. Cell lysis was performed in RIPA buffer, and all cells were scraped with a cell scraper and put into a 1.5 mL centrifuge tube. Next, the tube was placed on ice and shaken on a shaker for 30 min, followed by centrifugation of the samples at 4 °C for 10 min at 12,000× *g*. The supernatant was collected, and the total protein of the extracted cells was determined using the BCA method. Then, buffer was added, and the sample was boiled in boiling water for 10 min to denature the protein. A total of 20 µg protein was taken for SDS/PAGE electrophoresis, transferred to the NC membrane using a semi dry membrane converter, and then sealed with 5% skimmed milk powder at room temperature for 2 h. Following an overnight incubation with the primary antibody, the membranes were washed three times for 5 min each with TBST. Subsequently, they were incubated with the secondary antibody for 2 h at room temperature. Then, TBST was used to wash the membrane 3 times, and the target strip was exposed with ECL luminous solution. The following primary antibodies were used: β-actin (Beyotime, AF5003) and LATS2 (abcam, ab243657).

### 2.8. Determination of Triglyceride Content and Cholesterol

BMECs were cultured in 6-well culture plates, and each treatment was repeated 3 times. After 48 h, the triglyceride and cholesterol contents were determined. Cells were then washed with PBS followed by lysis buffer addition. Thereafter, the supernatants were collected into a 1.5 mL centrifuge tube and incubated at 4 °C for 15 min. The total protein concentration of the supernatants was quantified using a BCA protein assay kit (Thermo, Beijing, China), which served as an internal control for normalizing the lipid measurements. The supernatant was heated at 70 °C for 10 min and centrifuged at 2000 rpm for 5 min. The contents of triglyceride and cholesterol were determined using commercial assay kits (Triglyceride Assay Kit, St. Louis, MO, USA; Cholesterol Assay Kit, Sigma-Aldrich, USA), according to the manufacturers’ instructions. The standard curve was drawn, and the concentrations of triglycerides and cholesterol were calculated. The measured lipid concentrations were normalized to the total protein content, and the normalized data are presented in the results as relative levels compared with the control group.

### 2.9. Detection of Luciferase Activity

The potential target genes of miR-302c were predicted using Targetscan (http://www.targetscan.org) (accessed on 2 December 2025) and PicTar (http://pictar.mdc-berlin.de) (accessed on 1 December 2025) (Supplementary Table S3). Based on the comprehensive prediction results, LATS2 was a potential target gene of miR-302c. Using bovine LATS2 gene 3′-UTR as templates, the miR-302cbinding site region was designed and amplified. LATS2 gene 3′-UTR fragments amplified using PCR and psiCHECK-2 luciferase vector were digested by XhoI and NotI and then connected overnight at 4 °C under the action of T4 ligase. BMECs were inoculated into a 48-well plate and transfected when the cell density reached about 50,000 cells per well.

### 2.10. Data Analysis

The results of the real-time fluorescent quantitative PCR were analyzed using 2^−ΔΔCT^. Statistical analyses of different treatment groups were conducted using one-way ANOVA and SPSS 18.0, and the results were expressed as the mean ± standard deviation. *, *p* < 0.05 means a significant difference, and **, *p* < 0.01 means an extremely significant difference.

## 3. Results

### 3.1. Differential Expression Gene and GO/KEGG Analysis

**Figure 1:**
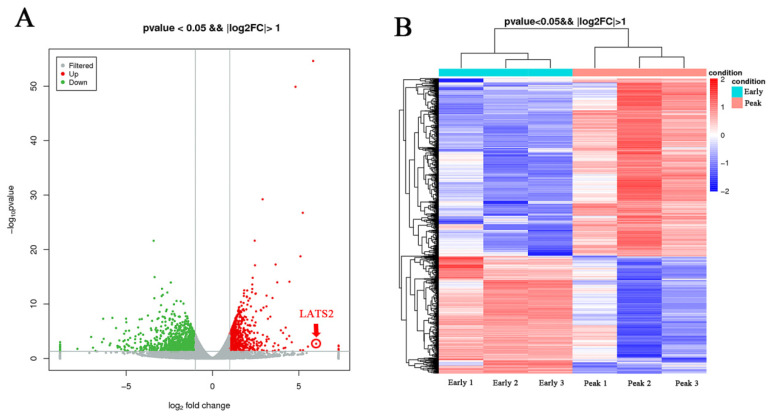
Differential expression gene analysis: (**A**) DEGs volcano diagram, with green dots indicating downregulated genes and red dots indicating upregulated genes; (**B**) DEGs clustering heatmap. Early lactation is the control group; peak lactation is the experimental group.

**Figure 2:**
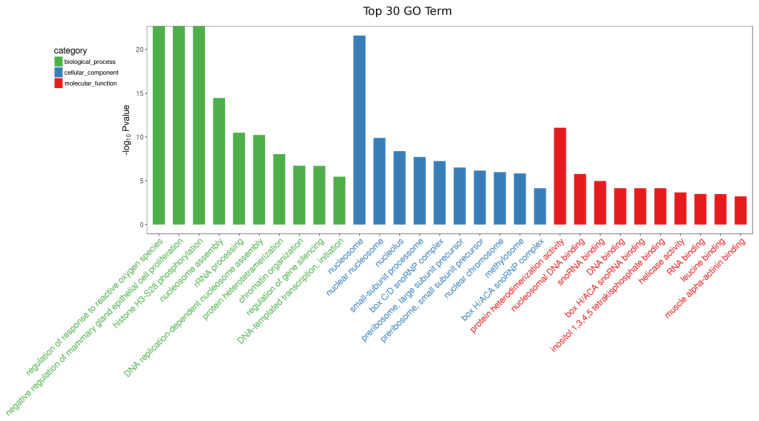
GO analysis of differentially expressed genes.

**Figure 3:**
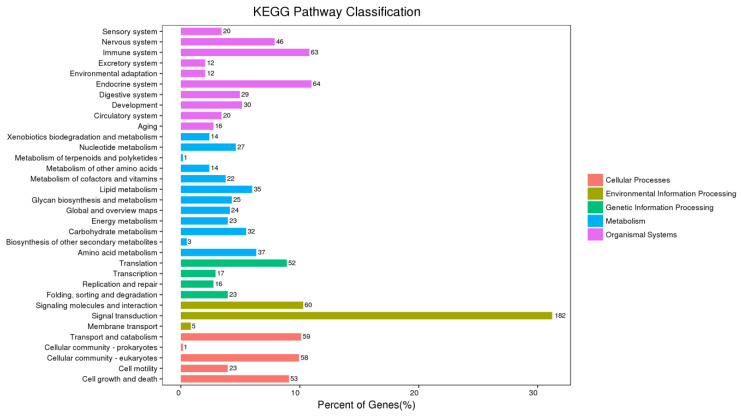
KEGG analysis of differentially expressed genes.

**Figure 4:**
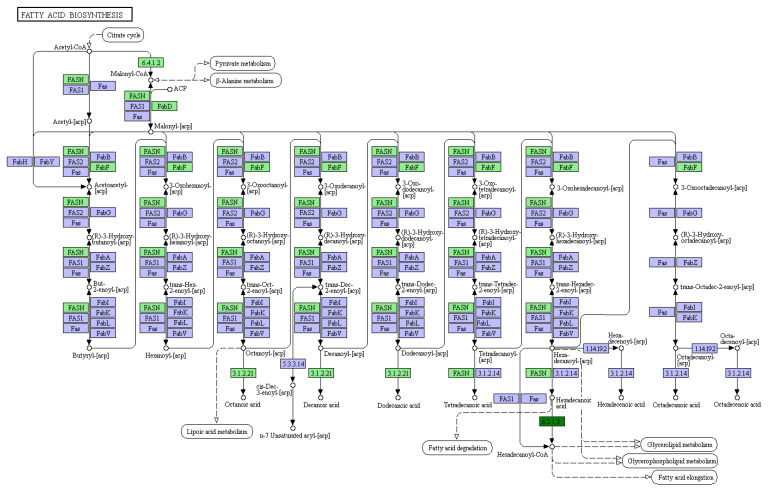
KEGG map of differentially expressed genes.

We undertook a comprehensive transcriptome sequencing analysis of dairy cow milk during both the early and peak lactation stages to elucidate the underlying molecular mechanisms. The results showed that a total of 1810 differentially expressed genes (DEGs) were identified, including 719 upregulated DEGs and 1091 downregulated DEGs, with early lactate as the control group and peak lactate as the experimental group (Figure 1). LATS2 was significantly upregulated (log2 fold change 5.53, *p* < 0.05) during the peak lactation period compared with the dry period and was therefore selected for subsequent analysis (Table S4). Subsequently, GO analysis was performed on the DEGs, and the results showed that the downregulated DEGs were mainly concentrated in biological processes such as the regulation of the response to reactive oxygen species, negative regulation of mammary gland epithelial cell proliferation, nucleosome assembly, rRNA processing, chromatin organization, regulation of gene silencing, RNA binding, leucine binding, muscle alpha–actinin binding (Figure 2). The KEGG analysis results showed that the DEGs were mainly enriched in cell growth and death, signal transduction, signaling molecules and interaction, lipid metabolism, metabolism of other amino acids, nucleotide metabolism, xenobiotics biodegradation and metabolism, circulatory system, development, environmental adaptation, excretory system, immune system, etc. (Figure 3). Interestingly, the KEGG map analysis showed that they were closely related to fatty acid biosynthesis (Figure 4).

### 3.2. Transfection Efficiency of circRNA, miRNA, and siRNA

**Figure 5:**
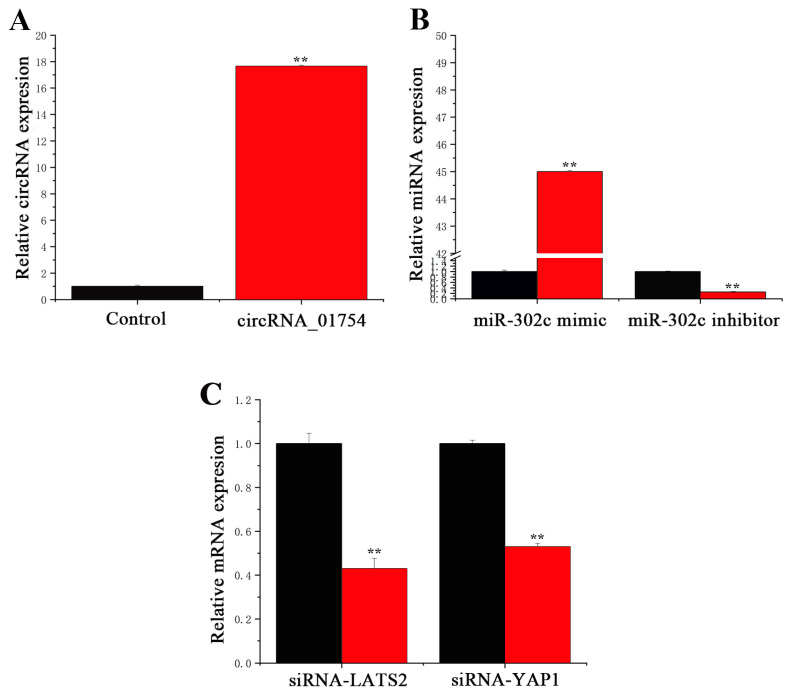
Transfection efficiency of circRNA_01754, miR-302c, siRNA-LATS2, and siRNA-YAP1. (**A**) Transfection efficiency of circRNA_01754. (**B**) Transfection efficiency of miR-302c. Black column: control group; red column: miR-302c mimic or miR-302c inhibitor. (**C**) MRNA expression level of LATS2 and YAP1. Black column: control group; red column: siRNA. **, *p* < 0.01 means an extremely significant difference.

To investigate the biological function of circRNA_01754 in BMECs, qPCR was used to detect the expression of circRNA_01754 in BMECs; the overexpression vector (pcDNA-circ01754, Tables S5 and S6) had an expression level of approximately 17 times. qRT-PCR was used to verify the mimic and inhibitor of miR-302c. As demonstrated in Figure 5A, the expression level of the miR-302c mimic in BMECs was 45 times higher than that in the control group (NC mimic). Compared with the NC inhibitor control, transfection with the miR-302c inhibitor resulted in a 99.6% reduction in mature miR-302c expression levels. This demonstrates that the transfection efficiency of pcDNA-circ01754 and miR-302c mimic and inhibitor is high, which makes them suitable for subsequent experiments.

qRT-PCR was performed to verify the transfection efficiency of siRNA-LATS2 and siRNA-YAP1. Figure 5C shows a 60% reduction in the siRNA-LATS2 expression level in BMECs compared with the NC siRNA control group. As a result of treatment, the expression level of siRNA-YAP1 was 50% lower than that of the control group (NC siRNA). As a result, siRNA is highly efficient in transfecting cells, making it available for subsequent experiments.

### 3.3. miR-302c Specific Targeting of LATS2

**Figure 6:**
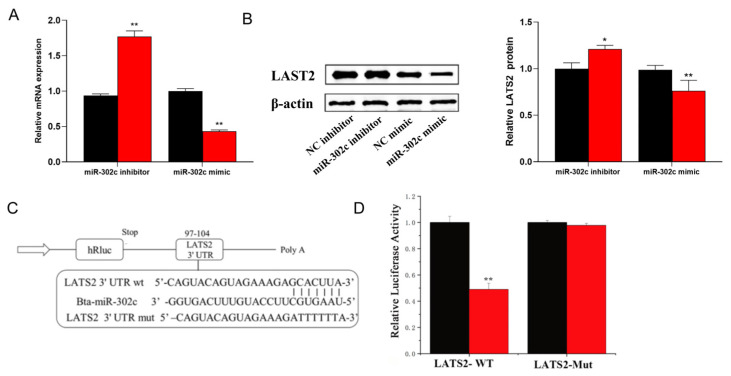
miR-302c specific targeting of LATS2. (**A**) MRNA expression level of LATS2. Black column: control group; red column: miR-302c mimic or inhibitor. (**B**) LATS2 protein expression level. (**C**) MiR-302c targets the LATS2 3′-UTR (97-104) site. (**D**) Activity detection of luciferase reporter vector fusion LATS2 3′-UTR. LATS2-WT: luciferase report vector wild-type LATS2 3′-UTR; LATS2-MUT: luciferase report vector mutant LATS2 3′-UTR. *, *p* < 0.05 means a significant difference, and **, *p* < 0.01 means an extremely significant difference.

LATS2 was selected as the research object to reveal the relationship and upstream regulation. The online software Target Scan 6.2 and miRNA analysis software DAVID (https://davidbioinformatics.nih.gov) (accessed on 20 June 2025) were employed. According to the analysis results, miR-302c was completely matched with the LATS2 gene 3′-UTR. As shown in Figure 6A, the miR-302c mimic downregulated the RNA expression level of the LATS2 gene, whereas the miR-302c inhibitor upregulated the LATS2 gene expression level. Overexpression of miR-302c resulted in LATS2 protein levels that correlated with the mRNA levels (Figure 6B). On the other hand, as shown in Figure 6C, the 3′-UTR of the LATS2 gene was combined with the miR-302c site. Based on this, the 3′-UTR fragment of the LATS2 gene was synthesized to determine whether miR-302c can directly target these sites. The DNA fragment containing the miR-302c target site was inserted into the psiCHECK-2 vector to generate a construct for subsequent cloning and identification. According to the luciferase report test, overexpression of miR-302c downregulated the activity of the 3′-UTR of the LATS2 gene in the wild-type, while in the mutant, its activity did not change (Figure 6D, Table S7).

### 3.4. circRNA_01754 Regulates LATS2 Expression Through Competitive Binding to miR-302c in BMECs

**Figure 7:**
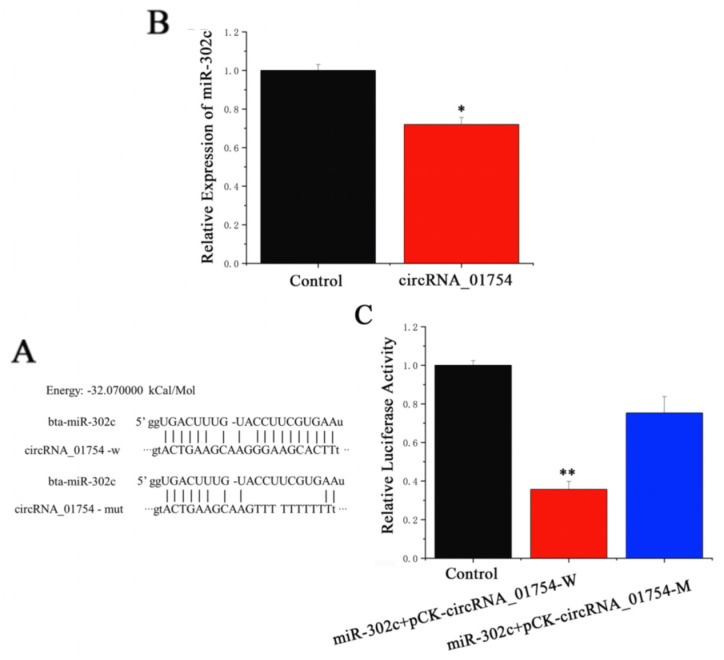
circRNA_01754 binding miR-302c to relieve its inhibition of LATS2. (**A**) The expression level of miR-302c; (**B**) RNAhybrid predicts the binding site between circRNA_01754 and miR-302c; (**C**) fluorescent enzyme activity detection. *, *p* < 0.05 means a significant difference, and **, *p* < 0.01 means an extremely significant difference.

To identify the regulatory factors of miR-302c, we used sequence analysis, which revealed a sequence binding relationship between circRNA_01754 and miR-302c (Figure 7A). Transfection of miR-302c mimic in BMECs revealed that miR-302c significantly reduced the expression level of circRNA_01754 (Figure 7B). To further validate that circRNA_01754 can bind to miR-302c, miR-302c mime and pCK-circRNA_01754 were co-transfected in HEK293T cells, and miR-302c significantly reduced the luciferase activity of pCK-circRNA_01754 (Figure 7C, Table S8). These results indicate that circRNA_01754 can competitively bind to miR-302c.

### 3.5. Functions of circRNA_01754 and miR-302c in BMECs

**Figure 8:**
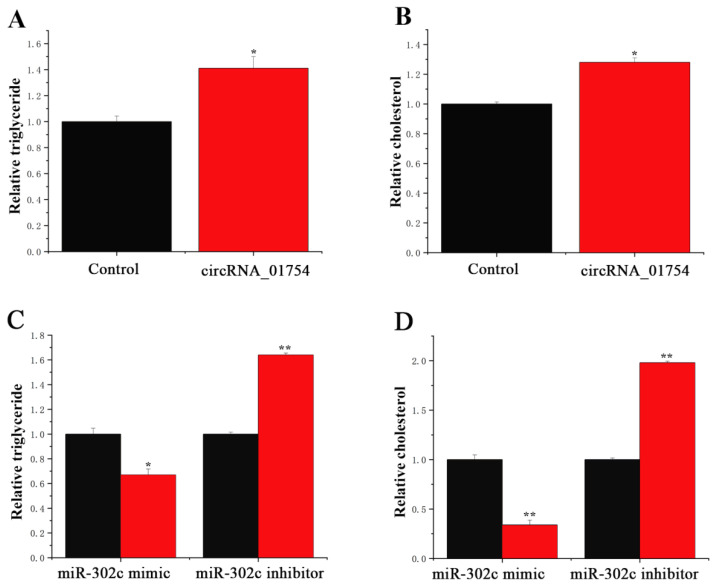
Functional research of circRNA_01754 and miR-302c.The black bars represent the control group, and the red bars represent the treatment group. (**A**) TAG level in BMECs treated with circRNA_01754. (**B**) Cholesterol levels in BMECs treated with circRNA_01754. (**C**) TAG level in BMECs treated with miR-302c mimic and miR-302c inhibitor. (**D**) Cholesterol levels in BMECs treated with miR-302c mimic and miR-302c inhibitor. All tests were repeated three times; the value was expressed as the mean ± standard error, * is *p* < 0.05, and ** is *p* < 0.01.

There are many small milk fat droplets in milk fat, which are formed by TAG in BMECs. In this study, we investigated the accumulation of lipid droplets and triacylglycerol (TAG) in BMECs under conditions of circRNA_01754 modulation. Compared with the control group, circRNA_01754 treatment significantly increased the difference in TAG contents by more than 1.4 times (Figure 8A). Nearly 1.2 times growth was found in the circRNA_01754 treatment. In addition, the TAG in BMECs was detected when miR-200a was overexpressed and inhibited. When treated with the miR-302c inhibitor, the TAG content was more than 1.6 times higher than that in the control group (Figure 8C), and a significant increase was observed in the difference in the cholesterol content (Figure 8D). This indicates that miR-302c can suppress milk fat metabolism in BMECs.

### 3.6. circRNA_01754 Can Regulate Intracellular Triglyceride Content by Adsorbing miR-302c in BMECs After siRNA-LATS2 Treatment

**Figure 9:**
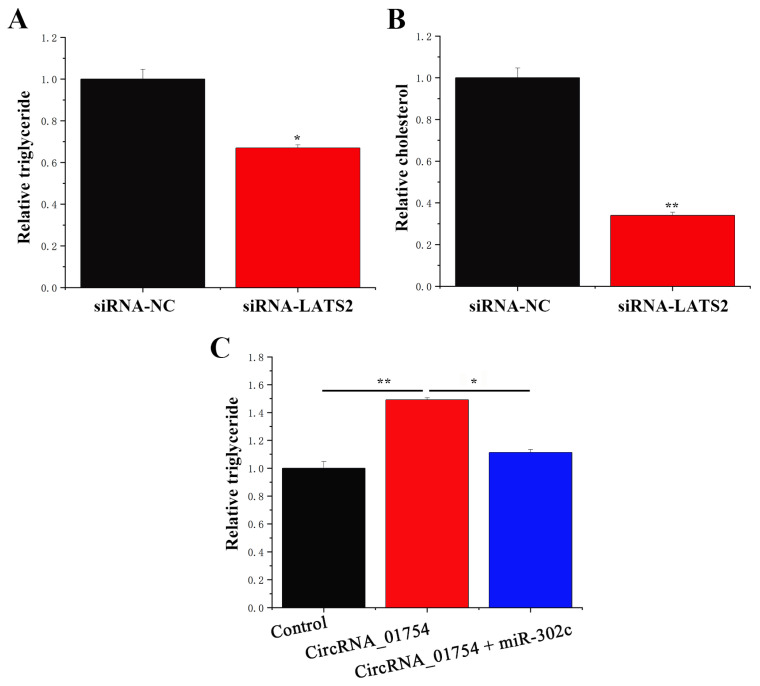
circRNA_01754 can regulate the intracellular triglyceride content by adsorbing miR-302c. (**A**) TAG level. (**B**) Cholesterol levels. (**C**) Triglyceride relative levels in the control, circRNA_01754, and circRNA_01754 + miR-302c groups. The value is expressed as the mean ± standard error, * represents *p* < 0.05, and ** represents *p* < 0.01.

The TAG content decreased by nearly 30% (Figure 9A), and the cholesterol content was reduced by around 60% (Figure 9B), indicating that LATS2 promoted milk fat metabolism in BMECs. The previous experimental results demonstrated that circRNA_01754 can competitively bind to miR-302c. Next, we examined whether circRNA_01754 affects triglyceride synthesis in cows by adsorbing miR-302c. This study co-transfected BMECs with pcDNA circRNA_01754 or (and) miR-302c mimics to investigate the effect of circRNA_01754 on triglycerides. The results showed that circRNA_01754 promoted triglyceride formation, but the overexpression of circRNA_01754 and miR-302c simultaneously reversed the effect of circRNA_01754 on BMECs. Thus, circRNA_01754 can regulate the intracellular triglyceride content in BMECs by adsorbing miR-302c.

### 3.7. miR-302c and LATS2 Synergistically Regulate YAP1

**Figure 10:**
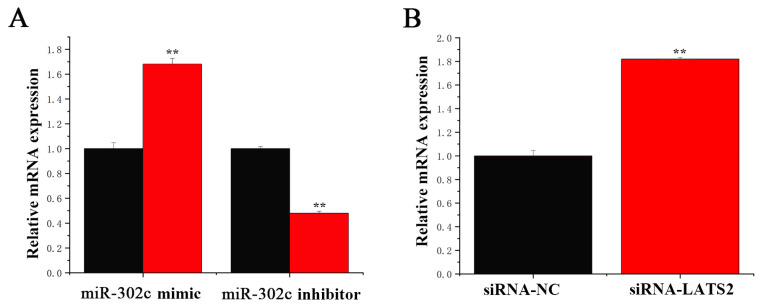
miR-302c and LATS2 synergistically regulate the YAP1. (**A**) YAP1 expression level. Black column: control group; red column: miR-302c mimic or miR-302c inhibitor. (**B**) MiR-302c expression level. Black column: siRNA-NC; red column: siRNA-LATS2. **, *p* < 0.01 means an extremely significant difference.

In this study, the miR-302c mimic downregulated the RNA expression level of YAP1 gene, whereas the miR-302c inhibitor upregulated the YAP1 gene expression level (Figure 10A). On the other hand, we also found that LATS2 inhibited the YAP1 mRNA expression (Figure 10B).

## 4. Discussion

### 4.1. miRNA in Animal Mammary Gland Research

The mammary gland is a type of white adipose tissue; and thus, it is similar to adipose tissue to a certain extent, but their development and function differ remarkably. We cloned 133 miRNAs from adipose tissue and 96 miRNAs from mammary gland tissue of dairy cows. Here, 23 and 9 miRNAs were specifically expressed in adipose tissue and mammary gland tissue, respectively. In addition, the copy numbers of miRNAs co-expressed in the two tissues were obviously different, indicating the specificity and significance of miRNAs involved in the fat metabolism of breast tissue. miRNAs have been studied in breast tissue, but little is known about how they regulate milk fat metabolism. Sequencing and microarray technologies have been widely used to establish miRNA expression profiles in the mammary gland across different lactation stages. Chen et al. identified 3096 differentially expressed miRNAs in bovine mammary gland tissue, with miR-141, miR-200a, miR-200b, and miR-200c being associated with lipid metabolism [16]. The research revealed several differentially expressed miRNAs. Several miRNAs screened were speculated to be associated with lactation function and mammary gland development. The maturation of miRNA has undergone two forms: pri-miRNA and pre-miRNA, which are complementarily paired with specific bases of the target gene mRNA, leading to mRNA degradation or translation inhibition, thus regulating genes and participating in biological activities [17]. The mature sequences of each member of the miR-302s gene cluster are highly conserved, especially the seed sequences. On the one hand, its conservation indicates the importance of the functions of these miRNA genes. On the other hand, it indicates that the members of gene clusters cooperate and complement each other regarding function, jointly playing a role in regulating signal network pathways [18]. Moreover, this regulatory mode is more complex and efficient than that of a single miRNA. There is a complex interaction between miR-302/367 and the pluripotency genes of pluripotent stem cells, which provides a guarantee for the long-term stable proliferation of pluripotent stem cells in vitro and reveals its potential important role in somatic cell reprogramming [19]. The research on miR-302c in cow milk fat is still lacking, and no relevant reports have been found. This study found that miR-302c inhibits triglycerides and cholesterol in bovine mammary epithelial cells; hence, it fills the gap in the research of miR-302c in the dairy industry.

### 4.2. Hippo Signaling Pathway Affects Milk Fat Metabolism in Dairy Cows

The mammary gland is a complex secretory organ composed of diverse cells: epithelial cells, fat cells, fibroblasts, and immune cells growing from the nipple to the fat pad [20]. As an important cornerstone of China’s dairy industry, dairy cows have considerable research value. Milk fat is an important index of milk quality, and its composition and content determine the nutritional value of milk [21]. Breast fatty acids of ruminants primarily come from two sources: de novo synthesis and blood intake. A lot of attention has been paid to the synthesis of milk fat and the formation and secretion of fat droplets. Triglycerides are important components of milk fat, and their biosynthesis has been shown to be regulated by a variety of genes, indicating their promising research potential [22]. As shown in a prior report, miR-181b can regulate LATS1, YAP1, and other genes on the Hippo signaling pathway by itself, as well as its target gene Insulin receiver subtract 2 (IRS 2), and inhibit TAG synthesis, thereby finally reducing the level of milk fat [23]. The transcription factor TEAD functions with YAP, a downstream protein in the Hippo signaling pathway, to regulate cell proliferation and the inhibition of apoptosis [24]. As evidenced, YAP1 inhibits PPARγ, a key factor in fat formation that can inhibit adipogenesis and differentiation. Overexpression of PPARγ can regulate the Stearoyl Coenzyme A desaturase 1 (SCD1), directly binding to it. The results of this study demonstrated that YAP1 can inhibit the metabolism of milk fat in cow mammary gland cells, which is consistent with previous findings.

Milk fat percentage and milk protein percentage are important milk production traits, and key genes related to milk production traits are enriched in the Hippo pathway [25]. Transcriptional profiling of two different physiological states of the yak mammary gland using RNA sequencing. Therefore, these findings suggest that the Hippo/YAP signaling pathway regulates the synthesis of milk fat and milk protein, thereby influencing milk production traits. Triglycerides account for approximately 98% of the total lipids in milk, and activating YAP promotes the accumulation of lipids such as TG and cholesterol in liver cells. Knocking down the upstream negative regulator LATS2 of YAP significantly increases the triglyceride content. In this study, knocking down LATS2 inhibited triglyceride synthesis and accumulation, suggesting that LATS2 affects milk fat synthesis in BMECs. Milk fat synthesis is regulated by a complex signaling network, and SREBPs and PPARγ are important factors in fat synthesis [26]. LATS2 can activate SREBP1 and PPARγ, stimulating fat production and tissue growth [27]. The target gene SCD of SREBP1 inhibits fatty acid oxidation by promoting YAP1 expression and promotes the synthesis of membrane phospholipids, cholesterol esters, and triglycerides. FABP3, as a target gene for SREBP1 and PPARγ, is involved in fatty acid transport and fat deposition [28]. Our study determined that LATS2 inhibits the expression of YAP1 in BMECs. We speculate that the LATS2/YAP1 pathway is likely to be involved in the metabolism of cow milk fat, and our research results confirm this point.

### 4.3. circRNA_01754 Regulates Milk Fat Metabolism in Dairy Cows’ Mammary Glands

Accumulating evidence indicates that circRNAs are involved in regulating key biological processes in livestock, including growth, development, reproduction, and health, with particularly important roles emerging in lactation [29]. Numerous circRNAs associated with the physiology and pathology of lactation have been identified in the mammary glands of diverse species, including humans, cattle, sheep, goats, sows, and mice [30,31,32] Developmental Regulation of circRNAs in Normal and Diseased Mammary Gland: A Focus on circRNA-miRNA Networks. Research has found that there are a large number of circRNAs in the mammary gland tissue of dairy cows [33]. With changes during lactation, the expression level of circRNAs in the mammary gland also changes. Research has demonstrated that the expression level of circCSN1S1 is positively associated with the milk yield, and it regulates casein secretion by targeting miR-2284 [34]. In another study, high-throughput sequencing of bovine mammary gland tissue from early and peak lactation stages revealed that the expression of circ09863 is lactation-stage-dependent. Functionally, circ09863 promotes triglyceride accumulation and increases the proportion of unsaturated fatty acids in milk by sponging miR-27a-3p [35]. Numerous circRNAs have been implicated in milk fat metabolism. For instance, circRNA_007873, circRNA_010763, and circRNA_015622 have been found to significantly promote the transcription of milk fat synthesis-related genes. Furthermore, comparative analysis of mammary gland tissues from Small-Tail Han sheep and High Mountain Merino sheep revealed 4000 and 4039 circRNAs, respectively, with 33 circRNAs identified as significantly differentially expressed between the two breeds [36].

So far, there have been few studies on circRNA in breast tissue, especially in agricultural animals, with reports only on cows, sheep, and pigs. In summary, these studies demonstrate that circRNAs play key regulatory roles in animal mammary gland development, milk secretion, and the synthesis of milk nutrients. However, the exploration of the functions and mechanisms of circRNA is still in its infancy and urgently needs to be studied. This experiment selected miR-302c that may bind to circRNA_01754 through bioinformatics analysis and determined its binding site. The dual-luciferase reporter assay verified the binding of circRNA_01754 to miR-302c and predicted the targeting of miR-302c to LATS2, validating its targeting relationship. miR-302c mimic is transfected into cells overexpressing circRNA_01754, and we verified the effect of miR-302c on cell triglycerides through a triglyceride assay. miR-302c alleviated the increased triglyceride secretion activity caused by the overexpression of circRNA_01754.

## 5. Conclusions

**Figure 11:**
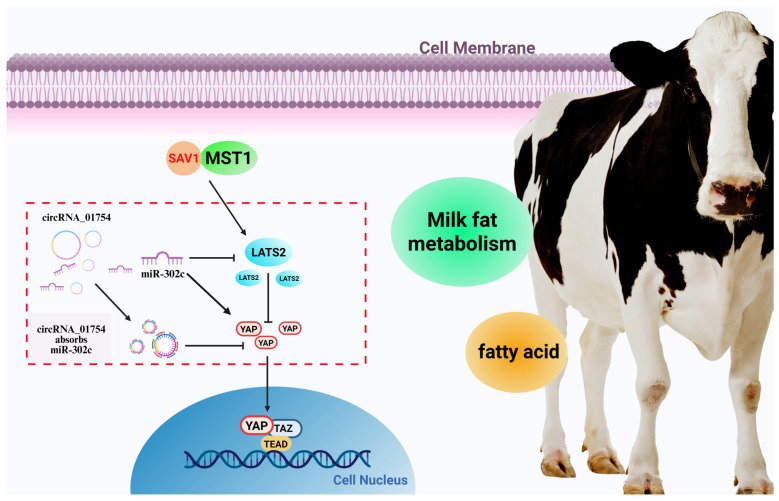
CircRNA_01754 regulates milk fat metabolism through the Hippo signaling pathway.

The composition and content of milk fat in milk have important impacts on its flavor nutrition, and BMECs synthesize most of the milk fat in breast tissue. Therefore, it is of great significance to study the molecular regulation mechanism of lipid metabolism in BMECs. In summary, circRNA_01754 functions as a molecular sponge for miR-302c, thereby upregulating the expression of its target gene, LATS2, through competitive binding. miR-302c can alleviate the enhanced triglyceride accumulation in BMECs caused by the overexpression of circRNA_01754 (Figure 11). This study provides theoretical and experimental evidence for understanding how the molecular mechanism of the circRNA–miRNA–mRNA pathway regulates lipid metabolism.

## Data Availability

All data in this manuscript have not been published. The materials, data, and associated protocols of this study are available from the corresponding author upon request.

## Supplementary Materials

The following supporting information can be downloaded at: https://www.mdpi.com/article/10.3390/ani15243606/s1.
